# A team approach to delivery of contextually relevant bioscience: encouraging student connections between tacit knowledge and new content acquisition

**DOI:** 10.1186/s12909-022-03513-5

**Published:** 2022-06-17

**Authors:** Mikaela Reynolds, Cristina Bowers, Holly Peters, Mathilde Klein, Zane Clayton, David Hagger, Ben McGarry, Elise Pelzer

**Affiliations:** School of Biomedical Sciences, Faculty of Health, Queensland University of Technology, 2 George Street, 4001 Brisbane, Queensland Australia

**Keywords:** Anatomy and physiology, Scaffolded learning, Cognitive load, Nursing, Education

## Abstract

**Background:**

Bioscience is essential knowledge for nursing practice and is an important component of undergraduate nursing education, however students commonly feel anxious about studying the subject. The purpose of this study was to develop appropriately scoped contextually relevant bioscience lesson resources to enhance student engagement and performance and reduce attrition and unit failures over a sustained period.

**Methods:**

Participants included students enrolled in the core bioscience unit for an undergraduate Bachelor of Nursing degree from a central campus and a widening participation (WP) campus. From 2016 to 2018, unit learning resources were progressively revised to include a structured learning and teaching manual, signposted lectures, and digital resources. Online surveys and formal institutional data collection metrics were used to assess the impact of the changes to unit learning resources.

**Results:**

Student attrition rates and failure rates for the unit were reduced over a two-year period across a diverse student cohort.

**Conclusions:**

Scaffolded and diverse learning materials support the success of undergraduate bioscience students by improving student engagement and reducing cognitive load.

## Background

Bioscience is an integral component of health science education, however, many students studying bioscience report feeling anxious and overwhelmed by the content and depth of knowledge required for their vocational destination [1–3]. Nursing students also state that bioscience units are more demanding than other nursing units and frequently report that whilst they are aware of the importance of bioscience to the role they will perform in healthcare, they often do not see direct links between the content taught in bioscience units and the clinical tasks that they will be required to perform in their jobs [4]. Although research has been conducted into strategies for teaching bioscience, it is evident that clinically relevant examples should be prominent in the course material [5–7].

Structured learning activities are pivotal in enabling students to develop metacognitive skills [8]. Scaffolded content enables students to progress through increasingly complex material. Creating a ‘guided tour’ highlighting familiar landmarks along the way is far more likely to result in active learning of unit content. The creation of a personalised learning environment has been linked with student success through enhancement of self-efficacy.

The purpose of this study was to develop appropriately scoped contextually relevant bioscience lesson resources to enhance student engagement and performance and reduce attrition and unit failures over a sustained period.

## Methods

### Aim

To develop contextually relevant and appropriately scaffolded bioscience lesson resources for nursing students from diverse backgrounds.

### Design

**Fig. 1:**
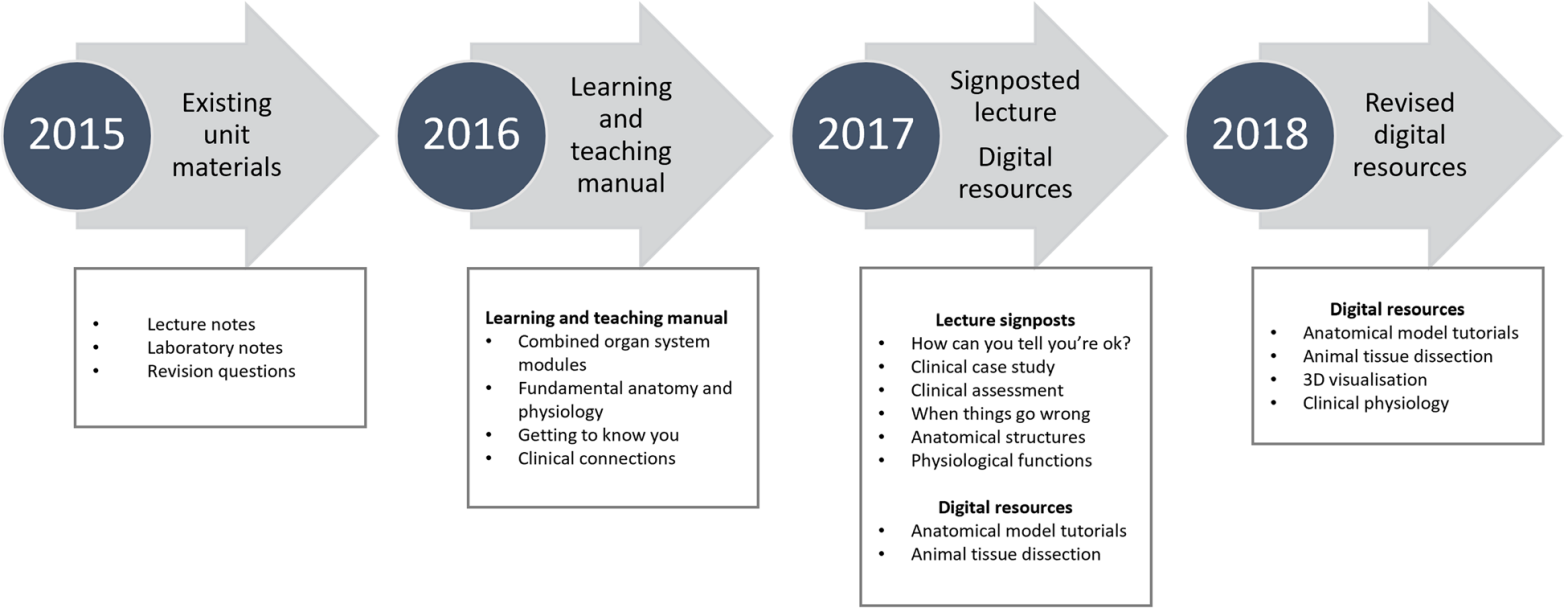
Redevelopment of unit learning and teaching materials. Unit learning materials were progressively revised and developed from 2016 to 2018 to include multimodal resource delivery and increased signposting to support student success. Updated changes are listed in the figure for the year in which the resources/changes were first introduced as student learning materials

Team teaching approach focused on contextual requirements of students. The unit content was repackaged progressively over a two-year period from 2016 to 2018 to incorporate specific signposting and scaffolding to reduce cognitive load (Fig. 1).

### Setting

Tertiary education institution in Australia. The teaching team comprised a staff group with clinical nursing experience, clinical exercise physiology experience, graduate qualifications specialising in advanced anatomy and physiology, and at least three years of undergraduate teaching in anatomy, physiology, nursing and microbiology units.

### Participants

Participants included students enrolled in the core bioscience unit for the undergraduate Bachelor of Nursing degree from the central campus and the widening participation (WP) campus. As part of the WP brief, improved access to the course was facilitated by lowering the entering metrics for these students compared to those enrolling to study at the central campus. The overall position (OP) cut-off was increased by 5-OP points, representing a considerably lower entry threshold for WP campus students.

### Redevelopment of unit learning and teaching materials:

#### Learning and teaching manual

In 2016, a learning and teaching manual was compiled, and interacting organ systems packaged together to reinforce global functions for students. For example, the Master control module coupled the nervous and endocrine systems as the soft and hardwired regulators of all cellular functions. Laboratory activities, questions and assessment were all included in the learning and teaching manual to ensure it was used as an evolving resource for students. Each module was signposted to include: *Fundamental anatomy and physiology*; *Getting to know you*; and *Clinical connections* sections. *Fundamental anatomy and physiology* covered essential anatomical structures and related those to the functions performed by cells, tissues, organs and organ systems as related to maintaining homeostasis. The *Getting to know you* section was aimed at highlighting tacit knowledge for students in the context of their lived experience, for example relating public speaking to the fight or flight response. The *Clinical connections* section was included to explicitly link the anatomy and physiology theory to the clinical practice of collecting observations and data from patients as part of a clinical assessment for diagnosis or monitoring of a health condition.

#### Lecture signposting

In 2017, the lecture structure was standardised and signposts included to guide students through each organ system. Lectures aimed to exploit student tacit knowledge through asking ‘How do you know you’re ok?’ and getting students to think about the head to toe assessment they perform unconsciously on everyone they interact with each day, and to focus familiar observations within the context of a specific organ system. For example, they could identify altered cardiovascular function if a person appeared flushed or pale. This provided a confident starting point for students, affirming that they were not learning about something completely new, rather, putting what they already knew into the context of the profession they were training for. The *Clinical case study*, *Clinical assessment* and *When things go wrong* sections highlighted the importance of regulating homeostasis and again provided tangible examples of knowledge already held by students such as dehydration due to exercise, and blood tests to measure deviations from a normal range. These sections often provided the ‘ah-ha’ moments for students where connections were made between the structure and function relationships underpinning the clinical measurement. Presentation of familiar examples reduced the cognitive loading experienced by students as each new topic was introduced.

#### Creation of digital resources

Additionally, in 2017, digital resources were planned through consultation between the teaching team, and storyboarding and scripting was undertaken during the planning phase to identify the appropriate scope for each presentation. Following the success of the initial videos generated by the teaching team, professional filming and editing services were sought.

The digital resources were designed to provide short foundation video tutorials describing the location of critical anatomical structures for major organ groups including the heart, the brain, the kidney and the eye with a corresponding instructional video demonstration of animal tissue dissection for these same organs. Three-dimensional (3D) interactive technology (Anatomage) was used to describe the anatomy and physiology of pulmonary ventilation and digestion. Video role plays were also developed to guide students through the stages of common tasks including electrocardiography, spirometry and simple exercises for supporting activities of daily living.

##### Student feedback

Student feedback was collected using institution implemented indicators of student satisfaction in the form of a centrally administered automated evaluation strategy comprising two formative (once early in semester and once at the end of the teaching period) surveys each comprising three Likert scale questions and one extended comment. Student satisfaction scores < 3.4 were considered as under-performing, were categorised as average for values ranging from 3.5 to 4.3 and were categorised as over-performing if > 4.1. A Googleform requesting specific feedback for the newly developed unit learning materials was also available to students via a web link. The Googleform was comprised of Likert scale questions such as “Did the brain model tutorial improve your knowledge of the structure and function of the organ?”, “Did the respiratory Anatomage tutorial improve your knowledge of the structure and function of the respiratory system in performing gas exchange?” and “Did the ECG tutorial improve your knowledge of the structure and function of the heart?” (with possible responses: strongly agree; agree; neutral; disagree; strongly disagree; I did not use this resource). The students were also asked to indicate which learning resources they utilised (by checking all that apply) and to rank the value of each of the learning resources to their understanding of the unit content from most helpful (1) to least helpful (18). Student response rates for the Googleform survey ranged from 16 to 19% during the study period. All student feedback was voluntary and anonymous.

## Results

From 2014 to 2017, the nursing cohort was spread across two campuses – the central campus and a WP campus. For the data collection period included in this study (2014–2017), the majority of the cohort were female (> 80%) and domestic (> 90%) rather than international. International student enrolments were only though the central campus; all WP enrolments were domestic students. School-leavers (students entering tertiary education directly after completing senior schooling) formed the larger proportion of all cohorts accounting for 46–72% of all students (Table 1). There were no differences between the campuses for non-school leaver numbers in 2014 or 2015, but for 2016 and 2017 there were significantly more non-school leaver students at the WP campus compared to the central campus (36% vs. 60% and 35% vs. 53% respectively, both *p* < 0.05). Non-school leaver numbers remained consistent across all four years for the central campus, but increased at the WP campus for 2016 and 2017. There were no significant differences across 2014 to 2017 between the central campus and WP campus for low socioeconomic status (SES), Aboriginal and Torres Strait Islander or disability student numbers (Table 1).

**Table 1:** Student demographic data for teaching periods from 2014–2017

	S1 2014	S1 2015	S1 2016	S1 2017
**TOTAL Unit Enrolments**	**478**	**536**	**603**	**511**
Domestic	94%	97%	95%	94%
International	6%	3%	5%	6%
Non school-leaver	51%	46%	48%	55%
Low-SES	17%	22%	23%	20%
Aboriginal and Torres Strait Islander	3%	3%	3%	4%
Disability	3%	3%	3%	4%

Student attrition rates were independent of campus for the period 2014 to 2017 (*p* > 0.05) (Table 2). Implementation of the first scaffolded change in content delivery (2016) resulted in a significant reduction in student attrition rates across all groups (*p* < 0.0001).

**Table 2:** Student attrition rates for both campuses for teaching periods from 2014–2017

Location	Intake	Admitted	% withdrawn	*p*-value
WP campus	S1 2014	117	11.9	*p* > 0.05
Central campus	S1 2014	368	12.7	
WP campus	S1 2015	153	13.7	*p* > 0.05
Central campus	S1 2015	386	11.1	
WP campus	S1 2016	145	4.8	*p* > 0.05
Central campus	S1 2016	392	3.5	
WP campus	S1 2017	121	2.4	*p* > 0.05
Central campus	S1 2017	389	3.3	

Failure rates were significantly increased in the WP campus compared to the central campus for the period 2014 to 2015 (*p* < 0.001 and *p* < 0.0001 respectively), but not for the period 2016 to 2017 (*p* > 0.05) (Table 3). Failure rates for the WP campus declined significantly from 2014 to 2015 to 2016 (*p* = 0.01) and remained constant from 2016 to 2017 (*p* > 0.05).

**Table 3:** Student failure rates for teaching periods from 2014–2017

Location	Intake	Total Students	% Failed	*p*-value
WP campus	S1 2014	94	27.6	*p* < 0.001
Central campus	S1 2014	353	10.4	
WP campus	S1 2015	117	26.5	*p* < 0.0001
Central campus	S1 2015	399	7.60	
WP campus	S1 2016	116	12.0	*p* > 0.05
Central campus	S1 2016	453	10.8	
WP campus	S1 2017	115	7.2	*p* > 0.05
Central campus	S1 2017	373	6.3	

Although the failure rate did not decline significantly in the central campus from 2014 to 2015, a trend towards a more normal distribution of higher grades is evident from 2016. The institution uses a 7-point grading scale (7 ≥ 85%; 6 ≥ 75%; 5 ≥ 65%; 4 ≥ 50%; 3 ≥ 40%; 2 ≥ 25%; 1 ≤ 24%). Significant differences were seen between the WP campus and central campus for grades 6 and 7 in 2014, grades 3 and 6 in 2015 and grade 6 in 2017 (Table 4).

**Table 4:** Distribution of grades for teaching periods from 2014–2017

Location	Intake	1	2	3	4	5	6	7
WP campus	S1 2014	3.4	8	11.5	39.1	21.8	9.2**	6.9*
Central campus	S1 2014	2	2.3	4.6	20.7	21.3	29.7	19.3
WP campus	S1 2015	4.3	4.3	17.9***	35.9	26.5	6.8**	4.3
Central campus	S1 2015	1.3	2.3	4	31.1	33.8	22.1	5.5
WP campus	S1 2016	2.6	3.5	5.2	58.3	20.9	8.7	0.9
Central campus	S1 2016	3.2	1.5	6.4	45.3	32.6	10.1	0.9
WP campus	S1 2017	8.5	2.5	4.2	36.4	33.1	13.6***	1.7
Central campus	S1 2017	4.5	1.6	2.9	31.6	32.4	22.6	4.3
* *p* < 0.05, ** *p* < 0.001, *** *p* < 0.0001								

Student evaluation scores were above average (> 4.1) at the central campus across all years, but increased from average to above-average and were maintained at an above-average level at the WP campus from 2016 onwards. There was a trend towards increased response rate for the WP campus from 2014 to 2017, but no real change in response rate for the central campus (Table 5).

**Table 5:** Student evaluation scores for teaching periods from 2015–2017

Location	Intake	Score	Response Rate
WP campus	S1 2014	4.4	21%
Central campus	S1 2014	4.4	28%
WP campus	S1 2015	3.8	28%
Central campus	S1 2015	4.3	25%
WP campus	S1 2016	4.6	30%
Central campus	S1 2016	4.3	31%
WP campus	S1 2017	4.5	34%
Central campus	S1 2017	4.4	28%

### Learning and teaching resource feedback

Anonymous feedback was collected via a Googleform in 2016 and 2017. The majority of respondents were female (84%) and school-leavers less than 20 years of age (57%). The remaining respondents were aged 21–25 years (14%) or 25 years to over 50 years (30%) and were non-school leavers.

Almost 90% of respondents had never studied bioscience before (86%). Of the students who responded to the online survey, the majority (77%) indicated that the digital resources helped them to see links between anatomical structure and physiological functions. Respondents also indicated that the digital resources improved their understanding of specific anatomy and physiology content (45%) and digital videos for animal dissection made them feel more comfortable before coming to the lab (64%).

**Fig. 2:**
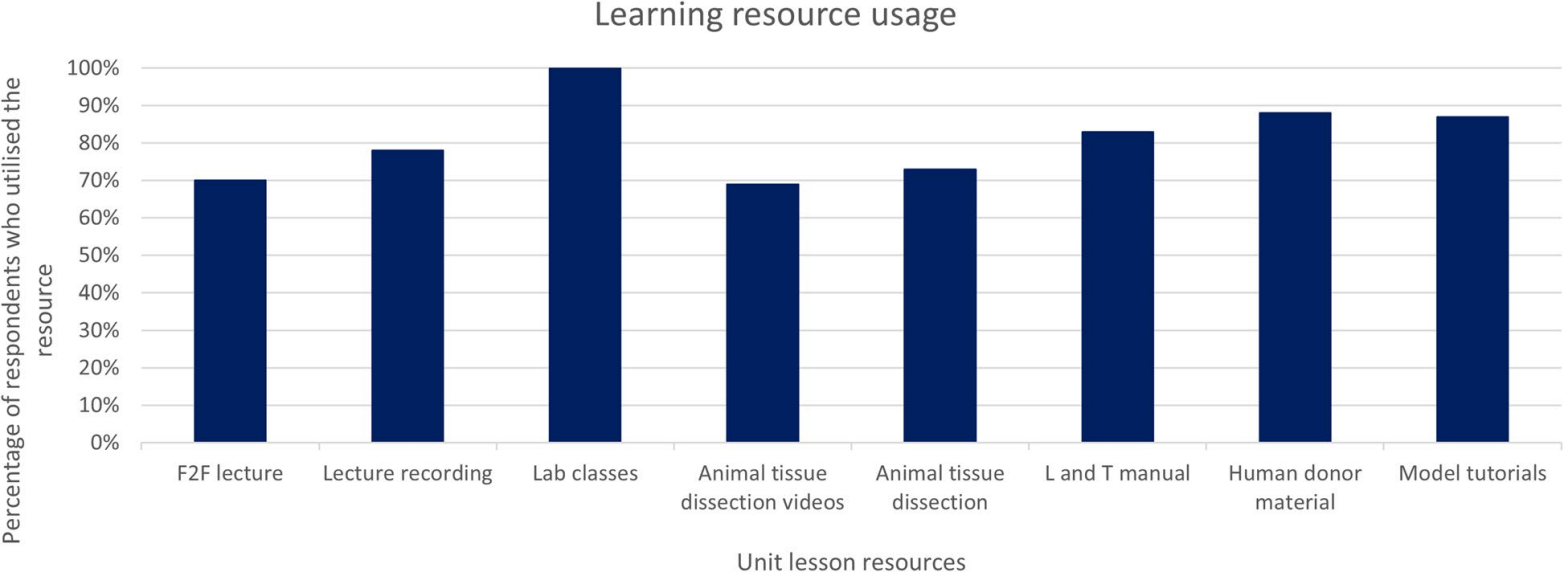
Learning resources most utilised in the unit (from total respondents). Students were provided access to an online Google form to ordinally rank all learning opportunities (lectures, laboratory class activities) and learning and teaching resources (learning and teaching manual, lecture recordings, digital media model tutorials and animal tissue dissection) from most to least utilised. The top eight most utilised resources are presented in the figure. Y-axis represents the percentage of respondents who utilised each resource

The most utilised learning resources were the laboratory classes, human donor material demonstrations, anatomical model tutorials (in-class demonstrations and digital resources), and the learning and teaching manual (greater than 80% of all respondents) (Fig. 2).

**Fig. 3:**
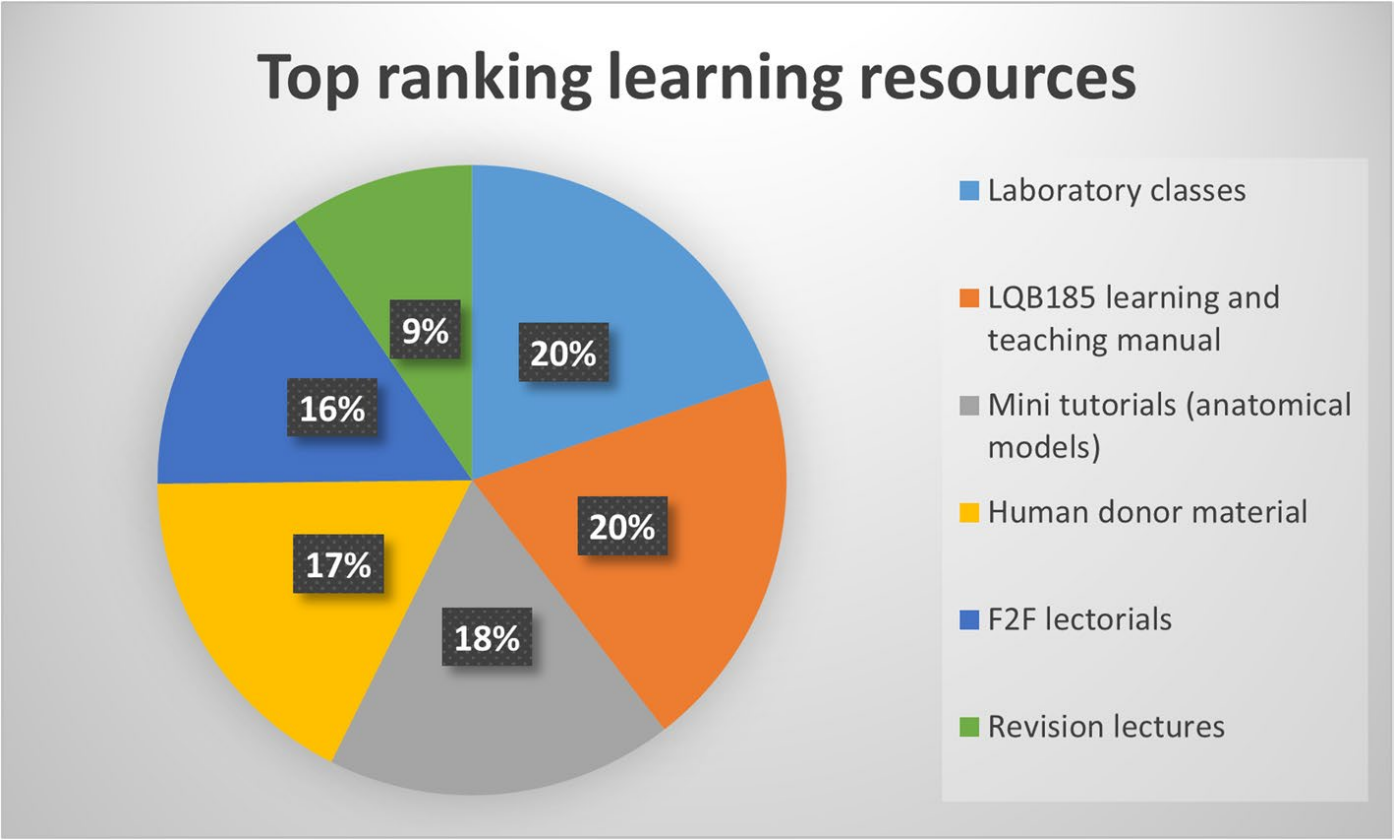
The most valuable learning resources according to the respondents. Students were provided access to an online Google form to ordinally rank all learning opportunities (lectures, laboratory class activities) and learning and teaching resources (learning and teaching manual, lecture recordings, digital media model tutorials and animal tissue dissection) from most to least useful in supporting their learning of anatomy and physiology content. All respondents ranked six of the top eight most utilised resources as most valuable for learning support

When asked to rank the most valuable learning materials in order, laboratory classes, the learning and teaching manual, anatomical model tutorials and human donor material were also placed at top of the list (Fig. 3).

## Discussion

Delivery of well-structured bioscience course materials results in sustained reduction of student attrition and failure rates. Our study confirmed that adequate scaffolding enables students to overcome cognitive overload, with the implementation of the first scaffolded change in content delivery (2016) resulting in a significant reduction in student attrition rates across all groups. Failure rates for the widening participation (WP) campus declined significantly from 2014 to 2015 to 2016, however, an increase in non-school leavers for the WP campus is also noted for these years and may have contributed to better overall outcomes for this cohort. Students with a limited level of education, as observed in our widening participation cohorts, often lack independent study skills and are easily overwhelmed by the scientific content associated with bioscience units [9]. High failure rates are consistently observed in such units [10]. The ability to deliver new knowledge, without creating cognitive overloading underpins the successful progression of students through undergraduate bioscience units. Anatomy and physiology encompasses not only new scientific knowledge, but also new language acquisition in the form of classical language word derivatives. By signposting connections between tacit knowledge and content we wanted students to understand within their vocational context, we were able to facilitate improved success. Commencing lectures with the same question each week ‘How can you tell you’re ok?’ gave students a consistent starting point and a low stakes discussion point to draw out their personal experience, to understand the value of their lived experience in studying bioscience, and to enable a confident start to the new topic. Getting students to make the connection between what is happening in the skin they are in, and the clinical measurements and observations that they will perform on future patients creates an impetus for deeper learning of the structural and functional relationships that regulate homeostasis in the human body.

A lecturer-focused approach to teaching often fails to support diverse student cohorts, promoting only superficial learning of content [8]. In contrast, methods that support collaborations with students facilitates deeper learning [11]. In support of the social constructivist theory, there has been a recent shift from traditional lecturer-focused approaches to collaborative group based approaches, encouraging students to gain motivation from participants in the learning environment [12]. Collaborative learning also enhances critical thinking, a skill that is essential to nurses who are faced with constant problem-solving tasks in the health-care system [13]. In this study, the laboratory environment was designed to encourage student collaboration, coupled with an appropriately structured learning and teaching manual to guide the students through the interactive learning activities.

Creation and delivery of engaging and relative learning materials that support variation in student learning styles are an important consideration for challenging units such as bioscience. In this study we created a suite of learning resources aimed at meeting the varied learning styles of our students. Understanding how students are learning, and the learning resources they preference should be carefully considered when designing learning and teaching materials [2]. The current generation employs digital technology and learning materials should support this platform to meet the student where they are comfortable. Further, the continual emergence of new technologies provides innovative alternatives to conventional classroom teaching [4]. Our data confirm that the digital media resources are perceived as valuable supplements to learning bioscience, but do not replace the tactile experiences available in laboratory classes. It is an expectation that when learning anatomy and physiology, nursing students spend time in the laboratory, engaging in a range of activities to fully grasp the content. For example, students engage with human donor material, plastic anatomical models, and operation of physiological equipment with corresponding interpretation of data output. Although these opportunities promote a rich learning environment, the students have a limited amount of time to spend observing the specimens in the laboratory sessions [14]. It is important to understand the relationship between structures when learning anatomy, therefore it can be difficult for students to engage in deep learning when they have limited time to individually manipulate and interact with the anatomical models. Evidence from our study and others confirms the importance of allocating sufficient time and resources to laboratory-based learning activities for bioscience students.

As observed in this study, student evaluation scores improved after the introduction of the additional learning resources. A study by Nicoll and Butler (1996) found that a lack of resources contributed to the anxiety experienced by students studying biology, and when changes were implemented, anxiety levels began to decrease [15]. Further, authentic learning was one of the key factors to nursing students’ satisfaction, indicating that for students to be successful in their learning, they need to be actively engaged with a focus on clinical skills rather than theory based learning [16]. Results from the surveys deployed in this study showed that students ranked the laboratory classes as the most valuable resource for their learning, where the students have the opportunity to be very hands-on and engage with teaching staff. The learning and teaching manual was also ranked equally as the most valuable resource, with the students appreciating the all-inclusive package, which provided fundamental anatomy and physiology questions and activities, and clinical connections. As studies have shown that nursing students experience higher levels of stress and anxiety when compared to non-nursing students, it is clear that additional support and engaging education experiences are required to maintain high student satisfaction [17, 18].

Nursification, defined as “the active association of a subject with nursing theory and practice” [19] (p1), reportedly increases motivation and enhances the process of effective learning of bioscience in nursing students [19]. One of the primary issues in the literature relates to nursing students lacking the understanding of how bioscience is relevant to nursing practice, and this results in knowledge of bioscience concepts being de-contextualised [20]. Ultimately, ensuring that nursing students understand the relevance of bioscience to nursing practice will positively impact clinical outcomes [21]. In order to advocate the importance of bioscience for nursing, this study introduced a learning and teaching manual which featured *‘Clinical Connections’* sections, as well as clinical case studies, clinical assessments and *‘When things go wrong’* sections in the lecture, continually introducing context to maintain interest and promote the relevance of bioscience to clinical practice. The results show that from 2016, when the improved learning and teaching manual was introduced, a significant reduction in student attrition and failure rates were observed indicating that the students felt more confident in their knowledge of bioscience.

## Conclusions

The introduction of contextually relevant bioscience learning resources for undergraduate nursing students resulted in increased engagement and a significant decline in fail rates and attrition rates. Additionally, student evaluation scores improved following the first change to the unit learning resources. Student feedback showed that the laboratory and the learning and teaching manual were the most valuable learning resources, followed by the mini anatomical model tutorials (in-class demonstrations and digital resources). This study demonstrates that the delivery of well-structured bioscience course materials helps to enable students to overcome cognitive overload and fosters confidence in student knowledge for an overall improved success.

## Data Availability

The datasets used and/or analysed during the current study are available from the corresponding author on reasonable request.

## Abbreviations

WP: widening participation
OP: overall position
3D: three-dimensional
SES: socioeconomic status
